# Supplementary material for: Unsaturated Fatty Acid Synthesis Is Associated with Worse Survival and Is Differentially Regulated by MYCN and Tumor Suppressor microRNAs in Neuroblastoma
Source: Cancers (Basel). 2024 Apr 21;16(8):1590. doi: 10.3390/cancers16081590 (PMC11048984; doi:10.3390/cancers16081590)
Supplement: Supplementary file 1 [file cancers-16-01590-s001.zip › cancers-2956050-supplementary.pdf]

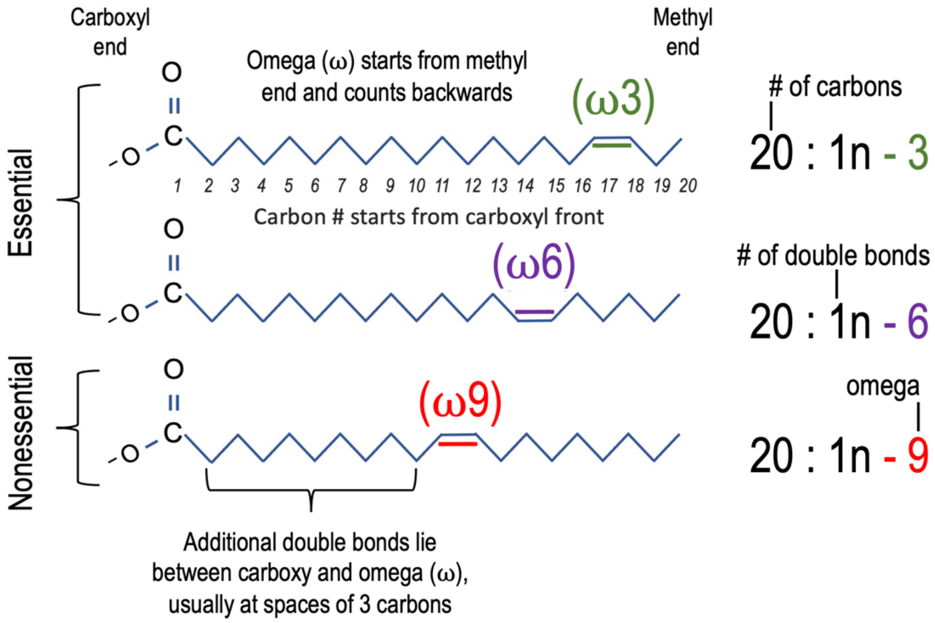

Supplemental Figure S1. Unsaturated fatty acid structure. Schematic of ω3, ω6, and ω9 fatty acid structures showing that carbon chains are numbered starting from the carboxy-terminus and that ω- double bonds are defined by distance from the terminal methyl group of the opposite end of the carbon chain. Nomenclature starts with number of carbons in the chain followed by a colon, then the total number "n" of double bonds in the chain, followed by a dashed number defining the ω class of the fatty acid.

Structure	Common name	DB location, (Δ)	Component
**Saturated**			
SFA	myristic (MA)	—	14:0
SFA	palmitic (PA)	—	16:0
SFA	margaric	—	17:0
SFA	stearic (SA)	—	18:0
SFA	arachidic	—	20:0
SFA	behenic	—	22:0
SFA	lignoceric	—	24:0
**Omega-9 (ω9)**			
MUFA	cis-hexadecenoic	Δ7	16:1ω9
MUFA	oleic (OA)	Δ9	18:1ω9
MUFA	gondoic	Δ11	20:1ω9
PUFA	8,11-eicosadienoic	Δ8,11	20:2ω9
HUFA	5,8,11-eicosatrienoic (Mead)	Δ5,8,11	20:3ω9
MUFA	erucic	Δ13	22:1ω9
HUFA	7,10,13-docosatrienoic (DTrA)	Δ7,10,13	22:3ω9
MUFA	nervonic	Δ15	24:1ω9
**Omega-7 (ω7)**			
MUFA	palmitoleic	Δ9	16:1ω7
MUFA	asclepic	Δ11	18:1ω7
**Omega-6 (ω6)**			
PUFA	linoleic (LA)	Δ9,12	18:2ω6
HUFA	gamma-linolenic (GLA)	Δ6,9,12	18:3ω6
PUFA	11,14-eicosadienoic	Δ11,14	20:2ω6
HUFA	sciadonic	Δ5,11,14	20:3ω6
HUFA	dihomo-gamma-linolenic (DGLA)	Δ8,11,14	20:3ω6
HUFA	arachidonic (ARA)	Δ5,8,11,14	20:4ω6
HUFA	adrenic (AdrA)	Δ7,10,13,16	22:4ω6
HUFA	osbond	Δ4,7,10,13,16	22:5ω6
**Omega-3 (ω3)**			
HUFA	alpha-linolenic (ALA)	Δ9,12,15	18:3ω3
HUFA	eicosapentaenoic (EPA)	Δ5,8,11,14,17	20:5ω3
HUFA	docosapentaenoic (DPA)	Δ7,10,13,16,19	22:5ω3
HUFA	docosahexaenoic (DHA)	Δ4,7,10,13,16,19	22:6ω3

Supplemental Table S1. List of fatty acids analyzed in this study. Table showing structure class (SFA = saturated fatty acid; MUFA = monounsaturated fatty acid; PUFA = polyunsaturated fatty acids with two double bonds (DB); HUFA = polyunsaturated fatty acids with three or more DB, common names and abbreviations, location of double bonds (location numbered from carboxy-terminus), and the component nomenclature. The "ω9" and "n-9" notations are interchangeable nomenclatures: e.g., 20:3ω9 = 20:3n-9 = Mead acid.

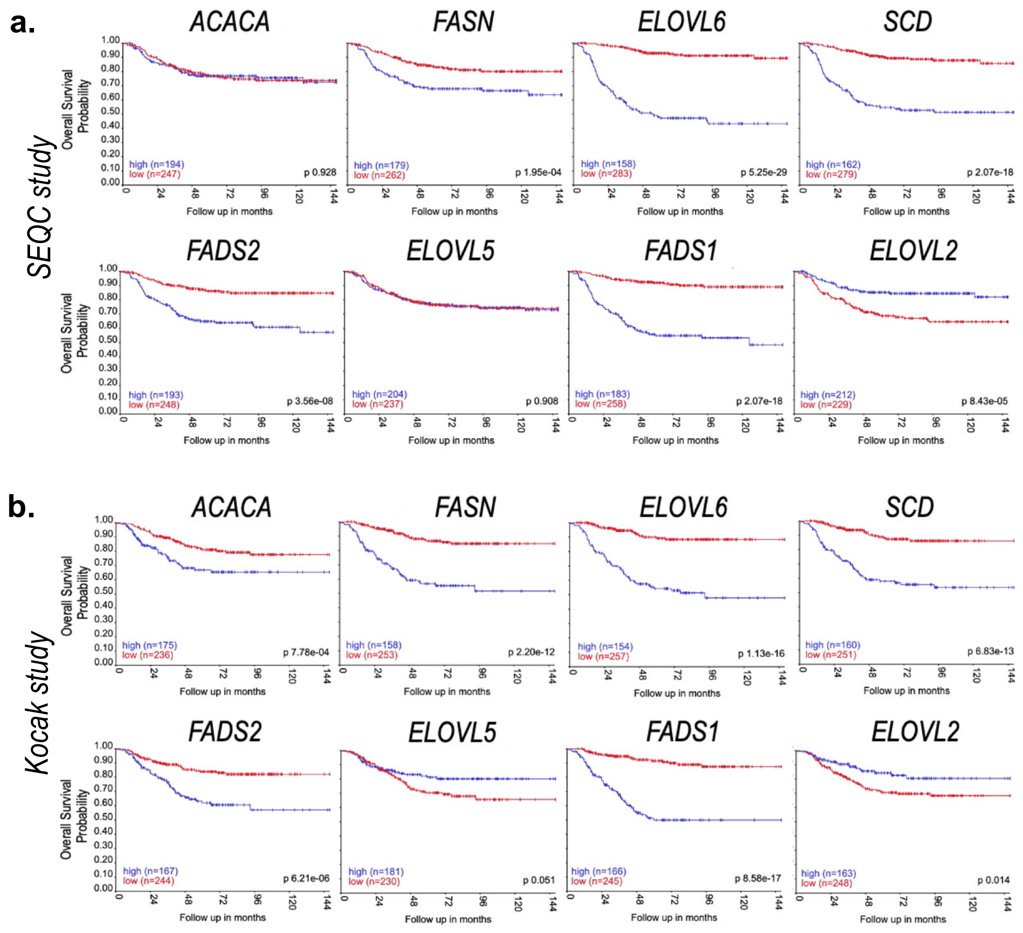

Supplemental Figure S2: Overall survival of NB patients relative to average U/FAS gene expression. Kaplan–Meier analysis of overall survival in high vs. low expressing NB patients (Stage 1-4) in the SEQC (n = 441) (**S2a.**) and Kocak (n = 411) (**S2b.**) studies. Significance was calculated using Bonferroni correction. Blue and red lines represent high and low gene expression, respectively. Patient numbers per group are in parentheses. Cut-off for high and low gene expression for Kaplan-Meier analysis was determined by average gene expression.

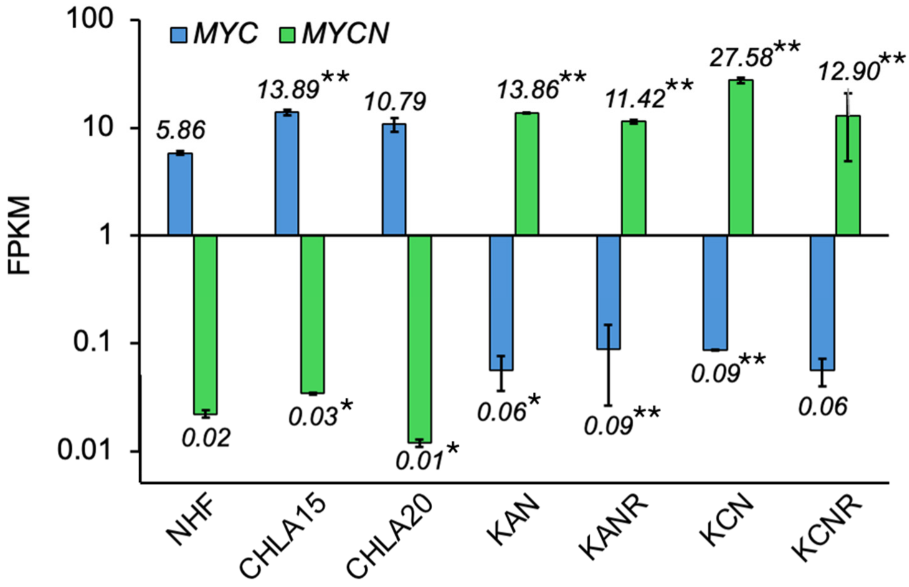

Supplemental Figure S3. FPKM gene expression levels of *MYC* and *MYCN* genes in NHF and human NB cell lines. (n = 3 sequencing rounds) Significance determined by t-test, **p*<0.05, ***p*<0.01.

Sample Name	Cell Type	Cell Line Designation	Sex	MYCN Status	Therapy Phase	Date Established	NB Cell Relationship	Research Resource Identifier (RRID)
NHF	Normal Human Fibroblast	BJ	male	—	—	2000	—	CVCL_3653
BE1	Neuroblastoma	SK-N-BE(1)	male	amplified	Dx	1972	paired	CVCL_9898
BE2	Neuroblastoma	SK-N-BE(2)	male	amplified	PD	1972		CVCL_0528
C15	Neuroblastoma	CHLA-15	female	non-amplified	Dx	1988	paired	CVCL_6594
C20	Neuroblastoma	CHLA-20	female	non-amplified	PD	1988		CVCL_6602
KAN	Neuroblastoma	SMS-KAN	female	amplified	Dx	1978	paired	CVCL_7131
KANR	Neuroblastoma	SMS-KANR	female	amplified	PD	1978		CVCL_7132
KCN	Neuroblastoma	SMS-KCN	male	amplified	Dx	1979	paired	CVCL_7133
KCNR	Neuroblastoma	SMS-KCNR	male	amplified	PD	1979		CVCL_7134

Supplemental Table S2. Human cell lines used in this study. Sample name is used for abbreviating graphs and corresponds to a unique cell line designation. NB cells were derived from tumors of HR patients at dates provided and are classified by sex, *MYCN* status, and therapy phase (Dx = at diagnosis; PD = during progressive disease). Paired NB cell sets were isolated from a single patient at diagnosis and during progressive disease. All cell lines were grown in RPMI media supplemented with 10% fetal bovine serum and were tested for mycoplasma before and during use.

Structure	Common name	Δ (DB location)	Component	NHF	BE1	BE2	C15	C20	KAN	KANR	KCN	KCNR
**Saturated**												
SFA	myristic (MA)	—	14:0	1.74 ± 0.10	1.49 ± 0.01	1.96 ± 0.05	2.32 ± 0.08	1.87 ± 0.05	2.33 ± 0.25	2.18 ± 0.31	3.29 ± 0.07	2.83 ± 0.01
SFA	palmitic (PA)	—	16:0	20.90 ± 0.37	19.12 ± 0.08	20.63 ± 0.06	24.22 ± 0.21	25.78 ± 0.07	26.74 ± 0.08	28.22 ± 0.90	23.09 ± 0.15	24.51 ± 0.25
SFA	margaric	—	17:0	0.75 ± 0.00	0.11 ± 0.02	0.14 ± 0.01	0.07 ± 0.01	0.14 ± 0.01	0.07 ± 0.03	0.19 ± 0.05	0.11 ± 0.04	0.17 ± 0.03
SFA	stearic (SA)	—	18:0	15.97 ± 0.35	16.96 ± 0.27	12.56 ± 0.11	12.37 ± 0.07	11.31 ± 0.04	10.52 ± 0.15	10.32 ± 0.49	14.02 ± 0.02	13.30 ± 0.01
SFA	arachidic	—	20:0	0.13 ± 0.00	0.12 ± 0.00	0.15 ± 0.02	0.16 ± 0.07	0.13 ± 0.02	0.20 ± 0.06	0.27 ± 0.08	0.09 ± 0.02	0.09 ± 0.02
SFA	behenic	—	22:0	0.45 ± 0.02	0.03 ± 0.02	0.15 ± 0.05	0.11 ± 0.04	0.27 ± 0.02	0.14 ± 0.00	0.07 ± 0.01	0.02 ± 0.00	0.09 ± 0.05
SFA	lignoceric	—	24:0	1.05 ± 0.01	0.04 ± 0.02	0.12 ± 0.00	0.17 ± 0.02	0.16 ± 0.06	0.07 ± 0.01	0.07 ± 0.01	0.06 ± 0.04	0.02 ± 0.00
**Omega-7 (ω7)**												
MUFA	palmitoleic	Δ9	16:1ω7	1.96 ± 0.04	0.59 ± 0.03	2.63 ± 0.01	2.43 ± 0.10	1.73 ± 0.25	1.79 ± 0.09	2.03 ± 0.02	1.53 ± 0.01	2.15 ± 0.12
MUFA	asclepic	Δ11	18:1ω7	6.82 ± 0.01	7.08 ± 0.12	9.22 ± 0.08	7.54 ± 0.04	7.56 ± 0.03	8.91 ± 0.03	9.31 ± 0.27	8.56 ± 0.00	7.63 ± 0.10
**Omega-9 (ω9)**												
MUFA	cis-hexadecenoic	Δ7	16:1ω9	2.37 ± 0.05	3.57 ± 0.00	3.75 ± 0.02	6.26 ± 0.02	5.40 ± 0.17	5.32 ± 0.01	4.77 ± 0.19	4.64 ± 0.02	4.83 ± 0.01
MUFA	oleic (OA)	Δ9	18:1ω9	20.98 ± 0.15	29.18 ± 0.27	32.42 ± 0.10	26.33 ± 0.08	24.31 ± 0.06	22.34 ± 0.05	22.03 ± 0.41	21.28 ± 0.19	26.24 ± 0.16
MUFA	gondoic	Δ11	20:1ω9	0.28 ± 0.03	1.35 ± 0.00	1.58 ± 0.02	1.39 ± 0.06	1.21 ± 0.09	1.45 ± 0.04	0.88 ± 0.07	1.12 ± 0.02	0.73 ± 0.03
PUFA	8,11-eicosadienoic	Δ8,11	20:2ω9	0.23 ± 0.03	0.11 ± 0.02	0.36 ± 0.03	0.85 ± 0.03	0.60 ± 0.04	0.12 ± 0.07	0.08 ± 0.00	0.24 ± 0.04	0.32 ± 0.06
HUFA	5,8,11-eicosatrienoic (Mead)	Δ5,8,11	20:3ω9	0.17 ± 0.01	5.36 ± 0.22	3.80 ± 0.10	5.23 ± 0.04	4.72 ± 0.02	4.91 ± 0.00	2.15 ± 0.44	3.93 ± 0.08	2.54 ± 0.04
MUFA	erucic	Δ13	22:1ω9	0.12 ± 0.03	0.07 ± 0.02	0.18 ± 0.01	0.20 ± 0.00	0.21 ± 0.05	0.10 ± 0.01	0.13 ± 0.01	0.05 ± 0.02	0.18 ± 0.05
HUFA	7,10,13-docosatrienoic (DTrA)	Δ7,10,13	22:3ω9	0.14 ± 0.01	5.96 ± 0.16	3.76 ± 0.08	3.76 ± 0.02	4.05 ± 0.01	5.75 ± 0.23	2.75 ± 0.43	5.42 ± 0.09	2.13 ± 0.07
MUFA	nervonic	Δ15	24:1ω9	1.61 ± 0.01	0.10 ± 0.08	0.68 ± 0.03	0.59 ± 0.02	0.85 ± 0.02	0.31 ± 0.04	0.21 ± 0.03	0.12 ± 0.04	0.24 ± 0.02
**Omega-6 (ω6)**												
PUFA	linoleic (LA)	Δ9,12	18:2ω6	2.11 ± 0.05	0.03 ± 0.00	0.39 ± 0.44	0.13 ± 0.01	0.23 ± 0.00	0.32 ± 0.25	0.13 ± 0.07	0.08 ± 0.03	0.07 ± 0.00
HUFA	gamma-linolenic (GLA)	Δ6,9,12	18:3ω6	0.30 ± 0.13	0.00 ± 0.00	0.00 ± 0.00	0.00 ± 0.00	0.00 ± 0.00	0.00 ± 0.00	0.00 ± 0.00	0.00 ± 0.00	0.00 ± 0.00
PUFA	11,14-eicosadienoic	Δ11,14	20:2ω6	0.09 ± 0.02	0.20 ± 0.06	0.69 ± 0.06	0.74 ± 0.02	0.54 ± 0.02	0.51 ± 0.02	0.18 ± 0.09	0.31 ± 0.02	0.07 ± 0.02
HUFA	sciadonic	Δ5,11,14	20:3ω6	0.00 ± 0.00	0.00 ± 0.00	0.00 ± 0.00	0.00 ± 0.00	0.00 ± 0.00	0.00 ± 0.00	0.00 ± 0.00	0.00 ± 0.00	0.00 ± 0.00
HUFA	dihomo-gamma-linolenic (DGLA)	Δ8,11,14	20:3ω6	1.90 ± 0.05	0.02 ± 0.02	0.11 ± 0.02	0.30 ± 0.04	0.47 ± 0.01	0.10 ± 0.05	0.18 ± 0.06	0.09 ± 0.01	0.12 ± 0.05
HUFA	arachidonic (ARA)	Δ5,8,11,14	20:4ω6	10.28 ± 0.44	3.62 ± 0.02	2.35 ± 0.01	2.72 ± 0.02	4.67 ± 0.02	3.90 ± 0.01	7.64 ± 0.58	5.04 ± 0.06	5.85 ± 0.02
HUFA	adrenic AdrA)	Δ7,10,13,16	22:4ω6	3.25 ± 0.07	2.13 ± 0.12	0.97 ± 0.11	0.79 ± 0.04	1.22 ± 0.06	0.71 ± 0.02	2.03 ± 0.16	3.46 ± 0.05	2.59 ± 0.06
HUFA	osbond	Δ4,7,10,13,16	22:5ω6	0.83 ± 0.03	0.51 ± 0.08	0.09 ± 0.01	0.20 ± 0.03	0.59 ± 0.03	1.55 ± 0.07	1.71 ± 0.18	0.34 ± 0.02	0.42 ± 0.04
**Omega-3 (ω3)**												
HUFA	alpha-linolenic (ALA)	Δ9,12,15	18:3ω3	0.36 ± 0.06	0.00 ± 0.00	0.00 ± 0.00	0.00 ± 0.00	0.00 ± 0.00	0.00 ± 0.00	0.00 ± 0.00	0.00 ± 0.00	0.00 ± 0.00
HUFA	eicosapentaenoic (EPA)	Δ5,8,11,14,17	20:5ω3	0.07 ± 0.05	0.00 ± 0.00	0.00 ± 0.00	0.00 ± 0.00	0.00 ± 0.00	0.00 ± 0.00	0.00 ± 0.00	0.00 ± 0.00	0.00 ± 0.00
HUFA	docosapentaenoic (DPA)	Δ7,10,13,16,19	22:5ω3	2.51 ± 0.13	0.91 ± 0.03	0.59 ± 0.03	0.22 ± 0.01	0.46 ± 0.00	0.18 ± 0.08	0.46 ± 0.04	1.37 ± 0.04	1.22 ± 0.13
HUFA	docosahexaenoic (DHA)	Δ4,7,10,13,16,19	22:6ω3	2.67 ± 0.19	1.35 ± 0.02	0.71 ± 0.01	0.90 ± 0.04	1.52 ± 0.05	1.66 ± 0.14	2.01 ± 0.14	1.73 ± 0.02	1.68 ± 0.12

Supplemental Table S3. NB cell line lipid levels master table. Fatty acids are ordered by structure and increasing component size. Numerical levels represent percent weight of a given fatty acid per overall weight (w/w, %) as determined by GC/MS (three biological replicates per cell line).

Gene	Gene ID	Ensembl ID	Ensembl length (nt)	RefSeq ID	RefSeq length (nt)
ACACA	31	ENST00000353139.5	2349	NM_198834.3	2324
FASN	2194	ENST00000306749.2	2245	NM_004104.5	805
ELOVL6	79071	ENST00000394607.3	5492	NM_024090.3	5492
SCD	6319	ENST00000370355.2	3901	NM_005063.5	3893
FADS2	9415	ENST00000257261.6	2890	NM_004265.4	1665
ELOVL5	60481	ENST00000370918.4	1741	NM_021814.5	1723
FADS1	3992	ENST00000350997.7	5184	NM_013402.7	2784
ELOVL2	54898	ENST00000354666.3	**3027**	NM_0177770.4	3022
MYCN	4613	ENST00000281043.3	917	NM_001293228.2	907
MYC	4609	ENST00000377970.2	1997	NM_002467.6	1993
MYCL	4610	ENST00000397332.2	1948	NM_001033081.3	1944
SDHA	6389	ENST00000264932.6	1269	NM_004168.4	662
ACTB	60	ENST00000331789.5	613	NM_001101.5	600
GAPDH	2597	ENST00000229239.5	224	NM_002046.7	201
DLK1	8788	ENST00000341267.4	3326	NM_003836.7	3328
ITGAV	3685	ENST00000261023.3	3610	NM_002210.5	3609
PTEN	5728	ENST00000371953.3	6458	NM_000314.8	6458

Supplemental Table S4. Table of human genes used to identify potential 3' UTR miRNA binding. The Ensemble ID and 3'UTR length are used to determine miRNA binding sites by TargetScan. Listed miRNA sites in *Table 1* were modified by limiting those sites to the RefSeq ID sequence and length, which are often shorter than the Ensembl sequences.

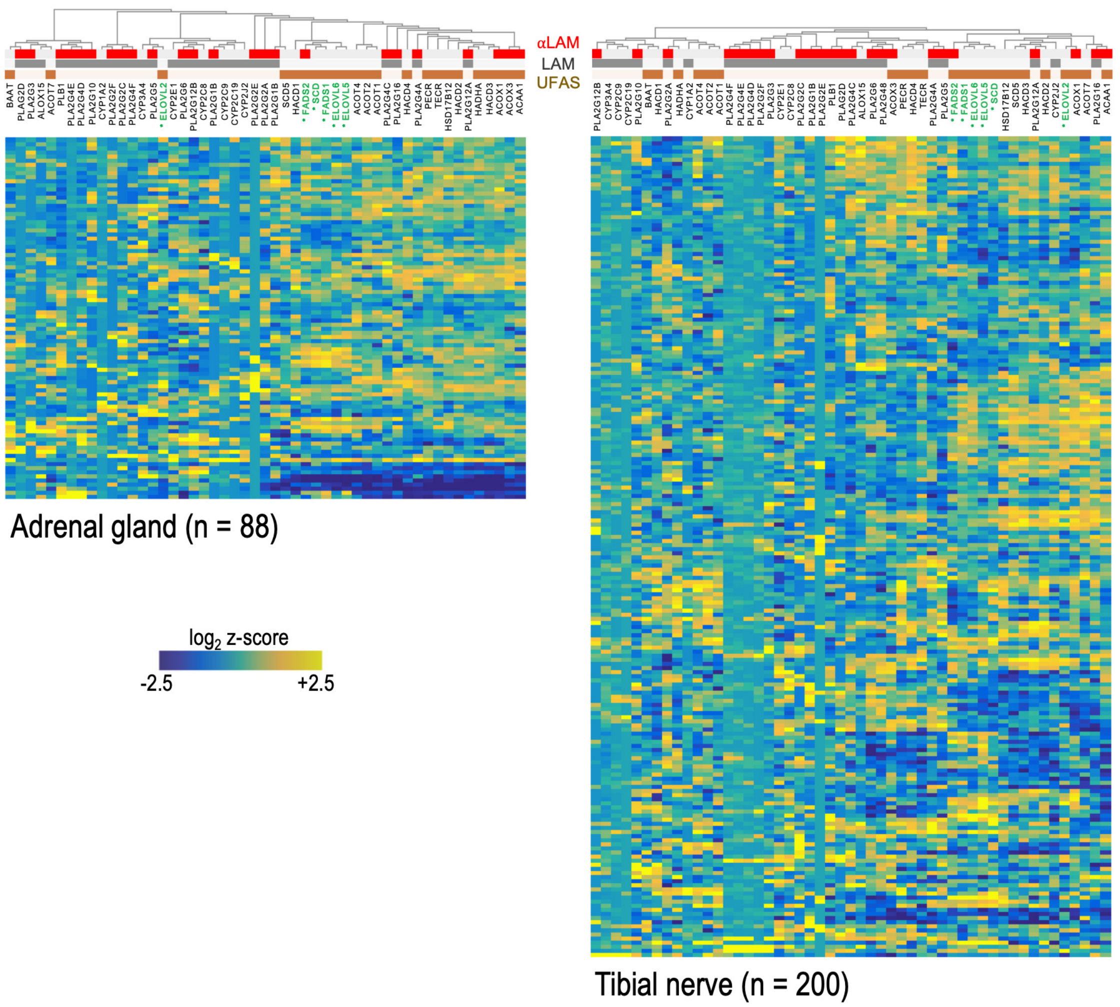

Supplemental Figure S4. Expression patterns in normal human tissues by KEGG gene set analysis. Heatmap of α-linolenic acid metabolism (α-LAM), linoleic acid metabolism (LAM), and unsaturated fatty acid synthesis (UFAS) gene expression comparing adrenal gland cells and tibial nerve cells to other body tissues. U/FAS pathway genes from *figure 1b* are titled in green and contain asterisks (*). Genes associated with α-LAM, LAM and U/FAS gene sets are identified by clustered red, grey, and brown bars, respectively. Expression data is presented as normalized values (log2 z-scores). (*GTEx Portal v8, protein coding*)

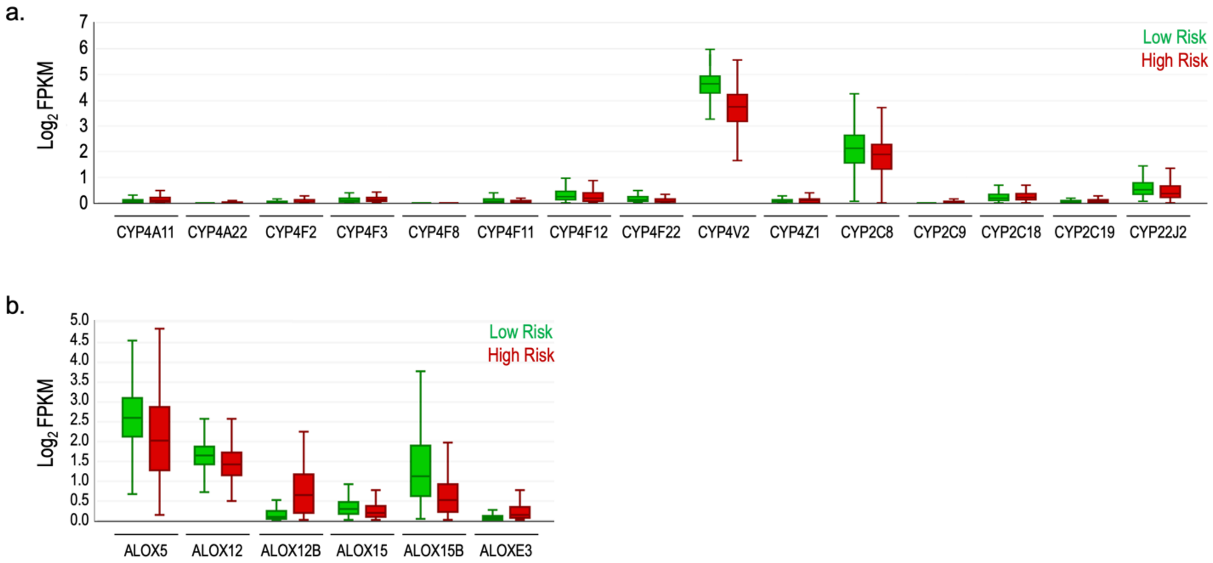

Supplemental Figure S5. Expression level comparisons of CYP450 and LOX genes in low-risk (LR) and high-risk (HR) NB patients. a-b. Expression levels of the *(a)* the fifteen human ω6 hydroxylating CYP450 and *(b)* the six human lipoxygenase (ALOX) genes in LR (green bars, n = 270) and HR (red bars, n = 171) NB. Gene expression is displayed for stage 1-4 NB patients by $\log_2$ FPKM values. (*GEO accession GSE62564*)

Figure	Key Word(s)	GEO Accession	Authors	PMID	R2 Internal Identifier
2a	NB	GSE62564	SEQC (Su)	25633159	ps_avgpres_gse49710geo498_ag44kcwolf
2b	NB	GSE62564	SEQC (Su)	25633159	ps_avgpres_gse49710geo498_ag44kcwolf
3a	NB	GSE62564	SEQC (Su)	25633159	ps_avgpres_gse49710geo498_ag44kcwolf
3b	NB	GSE62564	SEQC (Su)	25633159	ps_avgpres_gse49710geo498_ag44kcwolf
4b	*miR-22*	GSE83805	Bersani	27569217	n/a
4b	*miR-22*	GSE92599	Inwood	28987030	n/a
4b	*LIN28A (let-7)*	GSE53038	Kim	25479749	n/a
4b	*let-7*	GSE218501	n/a	not yet published	n/a
4b	*miR-124*	GSE67327	Shields	26655797	n/a
4c	NB	GSE62564	SEQC (Su)	25633159	ps_avgpres_gse49710geo498_ag44kcwolf
5a	Adrenal Gland	GSE3526	Various	N/A	ps_avgpres_adrenalglandns13_u133p2
5a	Adrenal Gland	GSE7307	Various	N/A	ps_avgpres_adrenalglandns13_u133p2
5a	Adrenal Gland	GSE8514	Various	N/A	ps_avgpres_adrenalglandns13_u133p2
5a	Trunk Neural Crest	GSE14340	Etchevers	19666486	ps_avgpres_gse14340geo5_u133p2
5a	NB	GSE16476	Versteeg	22367537	ps_avgpres_nbadam88_u133p2
5a	NB	GSE13136	Lastowska	17533364	ps_avgpres_nblastowska30_u133p2
5a	NB	GSE12460	Delattre	18553563	ps_avgpres_nbdelatre64_u133p2
5b	NB	GSE62564	SEQC (Su)	25633159	ps_avgpres_gse49710geo498_ag44kcwolf
5c	*MYC* (induction)	GSE14302	Chang	19211792	n/a
5d	MYCN (JQ1)	GSE107707	Iniguez	30537514	ps_avgpres_gse107707geo30_gpl18573b
5e	NB	GSE62564	SEQC (Su)	25633159	ps_avgpres_gse49710geo498_ag44kcwolf
6b	NB	GSE62564	SEQC (Su)	25633159	ps_avgpres_gse49710geo498_ag44kcwolf
7	NB	GSE62564	SEQC (Su)	25633159	ps_avgpres_gse49710geo498_ag44kcwolf
Sup S4a	NB	GSE62564	SEQC (Su)	25633159	ps_avgpres_gse49710geo498_ag44kcwolf
Sup S4b	NB	GSE62564	SEQC (Su)	25633159	ps_avgpres_gse49710geo498_ag44kcwolf

Supplemental Table S5. Publicly available GSE data sets used in this study. Data sets used for secondary analysis are organized by figure location and key words. Gene Expression Omnibus (GSE) accessions, study authors, PubMed Identifier (PMID), and R2 database internal identifiers are provided.
